# Toward the development of a novel non-RGD cyclic peptide drug conjugate for treatment of human metastatic melanoma

**DOI:** 10.18632/oncotarget.12748

**Published:** 2016-10-19

**Authors:** Boris Redko, Helena Tuchinsky, Tamar Segal, Dror Tobi, Galia Luboshits, Osnat Ashur-Fabian, Albert Pinhasov, Gabi Gerlitz, Gary Gellerman

**Affiliations:** ^1^ Department of Chemical Sciences, Ariel University, Ariel, Israel; ^2^ Department of Molecular Biology, Ariel University, Ariel, Israel; ^3^ Department of Computer Science, Ariel University, Ariel, Israel; ^4^ Department of Chemical Engineering, Ariel University, Ariel, Israel; ^5^ Department of Human Molecular Genetics and Biochemistry, Sackler School of Medicine, Tel-Aviv University, Tel-Aviv, Israel

**Keywords:** targeted drug delivery, human metastatic melanoma, Integrin αvβ3, non-RGD, conjugate

## Abstract

The newly discovered short (9 amino acid) *non-*RGD S-S bridged cyclic peptide ALOS-4 (H-cycl(Cys-Ser-Ser-Ala-Gly-Ser-Leu-Phe-Cys)-OH), which binds to integrin α_v_β_3_ is investigated as peptide carrier for targeted drug delivery against human metastatic melanoma. ALOS4 binds specifically the α_v_β_3_ overexpressing human metastatic melanoma WM-266-4 cell line both *in vitro* and in *ex vivo* assays. Coupling ALOS4 to the topoisomerase I inhibitor Camptothecin (ALOS4-CPT) increases the cytotoxicity of CPT against human metastatic melanoma cells while reduces dramatically the cytotoxicity against non-cancerous cells as measured by the levels of γH2A.X, active caspase 3 and cell viability. Moreover, conjugating ALOS4 to CPT even increases the chemo-stability of CPT under physiological pH. Bioinformatic analysis using Rosetta platform revealed potential docking sites of ALOS4 on the α_v_β_3_ integrin which are distinct from the RGD binding sites. We propose to use this specific non-RGD cyclic peptide as the therapeutic carrier for conjugation of drugs in order to improve efficacy and reduce toxicity of currently available treatments of human malignant melanoma.

## INTRODUCTION

Melanoma is the most deadly skin cancer, frequently associated with metastasis and poor survival prognosis [1]. According to the National Cancer Institute 76,690 people were diagnosed with melanoma in 2013 in the United States and 9,480 death accidents reported. Until now, systemic therapy for metastatic melanoma has been ineffective, but recent successes in the development of new therapies including mitogen-activated protein kinase (MAPK) pathway inhibitors, anti-Cytotoxic T-Lymphocyte Antigen-4 (CTLA-4) and Programmed cell death protein 1 (PD-1)/Programmed cell death 1 ligand 1 (PD-L1) 'blocking antibodies have all yielded promising results, enlarging the variety of therapeutic options for patients [1, 2].

Yet, the above mentioned therapies are still in development, and chemotherapy still remains the major treatment against metastatic melanoma [3]. The modest activity of single drug against metastatic melanoma has raised the possibility to use combinations of cytotoxic agents in order to improve outcomes (such as the combination of carboplatin and paclitaxel that is currently an NCCN cited “community standard” for treatment of patients with metastatic disease), but limited success has been achieved [4–8]. There have also been efforts to combine more than 2 cytotoxic agents, such as the Dartmouth regimen (combining cisplatin, dacarbazine, carmustine and tamoxifen), however it failed to show any statistically significant benefit [9, 10]. Furthermore, the cytotoxic effects of such drug “cocktail” treatments are severe and often intolerable by patients. Thus, new targeted drug delivery approaches are needed to overcome toxicological problems and improve efficacy.

One of the most effective techniques to achieve selective delivery of drugs is based on targeting over-expressed receptors on the surface of cancer cells. Integrins, for example, are a family of 24 known distinct cell surface receptors [11], whose variety of key roles in the neoplastic process has been thoroughly established [12]. Integrins are essential for tumor progression, and therefore are attractive targets for selective therapeutic intervention [13, 14] and drug delivery [15, 16]. Integrins are generally recognized by the “RGD tripeptide sequence” [17–19] and consequently many peptides bearing this recognition motif have been found to be effective ligands for the selective delivery of chemotherapeutics [15].

The use of peptides as targeting carriers is very intriguing due to their ease of synthesis, structural simplicity and high selectivity [20]. The most hopeful RGD peptide to date has been the selective α_v_β_3_ and α_v_β_5_ integrin antagonist cilengitide [15, 21], which reached Phase III clinical trials for the treatment of glioblastoma [22] and several other tumors [13, 22]. However, failure in demonstrating an adequate therapeutic and targeted effectiveness in clinical trials led to discontinued development [23]. Further complicating the use of RGD peptides as a chemotherapeutic delivery method is the finding that, paradoxically, some RGD-based inhibitors can *enhance*, rather than suppress, tumor progression. Reynolds *et al.* found that the continuous infusion of very low concentrations of RGD-mimetic inhibitors stimulates tumor growth and angiogenesis by promoting VEGF-induced endothelial cell migration [24]. Given these significant obstacles with RGD peptides, it is clear that alternative integrin-specific antagonistic peptide carriers are needed for targeted delivery of conjugated anticancer payloads to malignant melanoma cells for reducing toxicity and increasing efficacy.

Our research proposes to address these concerns by utilizing the newly discovered short (9 amino acids) *non*-RGD cyclic peptide ALOS4 (H-cycl(Cys-Ser-Ser-Ala-Gly-Ser-Leu-Phe-Cys)-OH) that binds to a *non*-RGD site upon integrin α_v_β_3_ [25]. Here we show that ALOS4 binds specifically human malignant melanoma cells both *in vitro* and *in vivo*. We were also able to conjugate the anti-cancer drug Camptothecin (CPT) to ALOS4 to achieve increased chemo-stability of CPT as well as specific internalization of CPT into human malignant melanoma cells to successfully induce DNA damage and tumor cell death. Moreover, bioinformatics analysis suggests that ALOS-4 *non*-RGD binding site in integrin α_v_β_3_ is between the Thigh domain and the Calf-1 domain of α_v_. Thus, ALOS4 has the potential to become a targeted drug carrier for treatment of human malignant melanoma.

## RESULTS

### The *non*-RGD cyclic peptide ALOS4 specifically binds human melanoma cells both *in vitro* and *in vivo*

**Figure 1:**
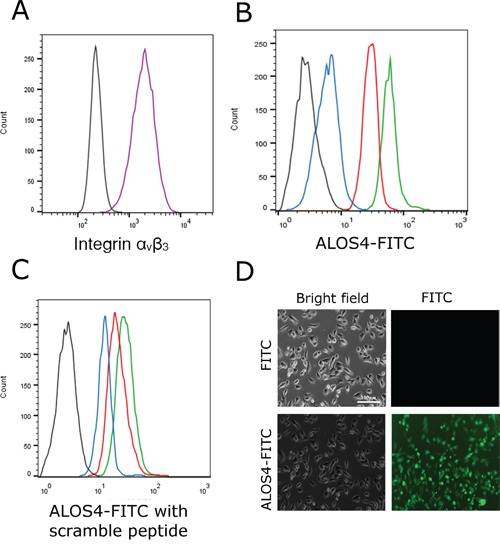
Binding of ALOS4-FITC conjugate to WM-266-4 human metastatic melanoma cells *in vitro* **A.** Expression of integrin α_v_β_3_ in WM-266-4 cells was evaluated by FACS analysis using FITC-conjugated integrin α_v_β_3_ antibody (black-control cells w/o an antibody, purple-cells incubated with FITC-conjugated integrin α_v_β_3_ antibody). **B.** Binding of ALOS4-FITC to the WM-266-4 cells at concentrations of 1μM (blue), 5μM (red) and 20μM (green). Auto-fluorescence of the cells is marked by a black line. **C.** Binding of 10 μM of ALOS4-FITC in the presence of the ''scrambled” peptide P1 at concentrations of 1μM (blue), 5μM (red) and 20μM (green). Auto-fluorescence of the cells is marked by a black line. **D.** Fluorescence of live WM-266-4 cells following incubation with 10 μM of FITC or ALOS4-FITC. Scale bar: 100 μm.

The *non*-RGD cyclic peptide ALOS4 has been shown to specifically bind mouse melanoma cells [25]. Usage of ALOS4 for therapeutics purposes will require specific binding to human melanoma cells. To evaluate ALOS4 ability to bind human malignant melanoma cells, the binding of FITC-labeled ALOS4 (ALOS4-FITC) to the human malignant melanoma cell line WM-266-4 was determined by FACS analysis (Figure 1). WM-266-4 cells express high levels of the integrin α_v_β_3_ in contrast to the non-malignant HEK293 cells (Figure 1A, Supplementary Figure S1). As expected, ALOS4-FITC specifically bound WM-266-4 cells in a concentration-dependent manner, while not affected by a non-specific peptide (Figure 1B-1D).

**Figure 2:**
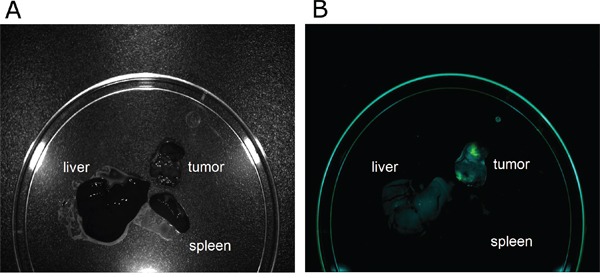
Tumor-specific retention of ALOS4-FITC WM-266-4 cells were injected subcutaneously into nude mice to induce a tumor. Mice bearing a 100 mm3 tumor were intravenously injected with ALOS4-FITC. 3 hours and 24 hours post injection, the tumor, the spleen and the liver were excised and evaluated for accumulation of ALOS4-FITC by Maestro™ *In-Vivo* Fluorescence Imaging System. The results of ALOS4-FITC accumulation after exposure for 3 hours and 24 hours were similar, therefore only the 24 hour time point is presented. **A.** Image of the excised organs. **B.** Unmixed composite image of ALOS4-FITC fluorescence.

Next, we evaluated if ALOS4 binding is strong and specific enough to target human malignant melanoma cells inside the animal. Nude mice bearing 100 mm^3^ tumors of subcutaneously injected WM-266-4 cells were intravenously administered with ALOS4-FITC. After 24 hours ALOS4-FITC was found to accumulate specifically in the tumor rather than in other organs (Figure 2). Thus, ALOS4 is able to specifically target malignant melanoma cells both *in vitro* and *in vivo*.

### Coupling ALOS4 to CPT

**Figure 3:**
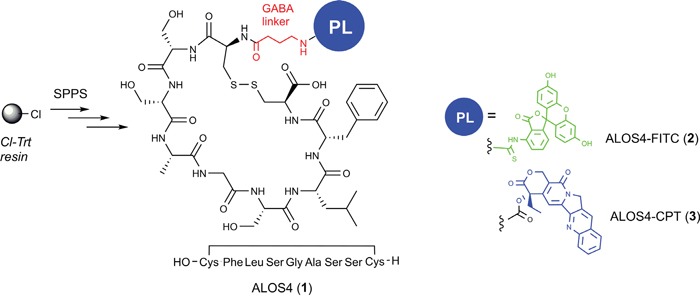
Schematic representation of synthesis of ALOS4 and its fusion to FITC and CPT All peptides were synthesized using solid phase organic synthesis (SPOS). Conjugations of FITC and CPT done via GABA linker.

Previous work has shown that although ALOS-4 can bind murine melanoma cells and inhibit their migration it does not affect cell viability and proliferation [25]. To add a cytotoxic activity to ALOS-4 we coupled it to the topoisomerase I inhibitor CPT [26]. ALOS4 peptide was synthesized using solid phase organic synthesis (SPOS). The cyclic peptide core was assembled on a 2-chlorotrityl chloride resin solid support using standard Fmoc solid phase peptide synthesis (SPPS) and coupled to CPT (Figure 3).

**Figure 4:**
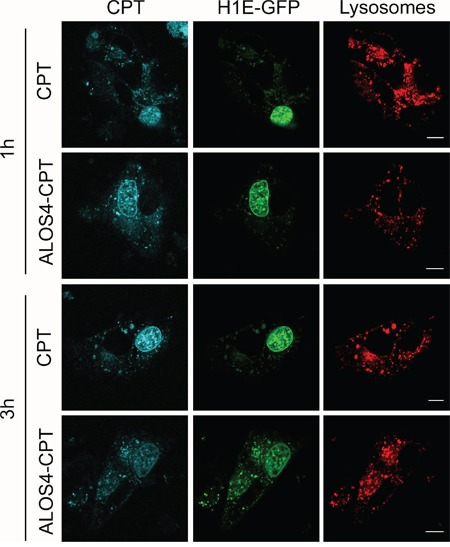
Successful internalization of CPT fused to ALOS4 Confocal images of live WM-266-4 cells over-expressing histone H1E-GFP (nuclear marker, green) that were incubated with CytoPainter Lysosomal Staining dye (lysosomal marker, red) and 10 μM CPT or ALOS-4 CPT for 1 hour and 3 hours. Scale bar: 10 μm.

Next, we confirmed that CPT can localize to the cell nucleus even when coupled to ALOS4. For that purpose, WM-266-4 cells were incubated with 10 μM of either CPT or ALOS4-CPT and the intracellular localization of CPT was monitored by confocal analysis of live cells. CPT can be detected due to its fluorescence properties, with an emission maximum at 435 nm [27]. As shown in Figure 4, incubation of the cells with the drugs for 1 h resulted in accumulation of CPT inside the nucleus and to some less degree in the lysosome. Interestingly, the intracellular localization of CPT that was pre-coupled to ALOS4 was similar to free CPT. Thus, CPT can still accumulate inside the nucleus when coupled to ALOS4.

### Chemo-stability tests of ALOS4-CPT conjugate *vs* free CPT

**Figure 5:**
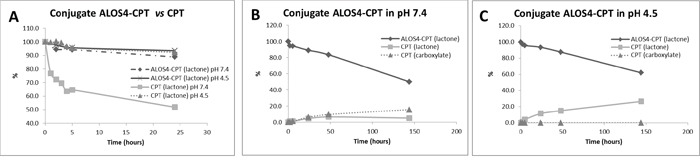
Chemo-stability of the free CPT and ALOS4-CPT conjugate at pH=4.5 and 7.4 **A.** Comparative stability of ALOS4-CPT *vs* free CPT. **B, C.** Chemo-stability of ALOS4-CPT and CPT release from the conjugate at pH 7.4 and 4.5, respectively. The drugs stability analyzed by LC-MS. All samples were injecting twice.

The stability of the anticancer peptide drug conjugate ALOS4-CPT in hemolytic and proteolytic environments is of significant importance for therapeutic applications. CPT, under such conditions, is prone to lactone ring opening to carboxylate followed by its deactivation [28]. Therefore, ALOS4-CPT conjugate should exhibit satisfactory stability until it is taken up by the cancer cells. We compared the chemo-stability of ALOS4-CPT conjugate to free CPT in two different pH values; a physiological pH of 7.4 and an acidic pH of 4.5, as the microenvironment in lysosomes [29, 30], while the solutions were incubated at 37°C. Aliquots were taken at selected time intervals and analyzed by LC–MS (Figure 5).

As expected, CPT alone went rapid opening of ring E: following 5 h of incubation at pH 7.4 almost 40% of the CPT underwent lactone ring opening to carboxylate. However, only 6% of CPT underwent lactone ring opening once conjugated to ALOS4 (Figure 5A). Prolonged incubations times revealed that only after more than 100 h of incubation at pH 7.4 40% of the ALOS4-conjugated CPT underwent lactone ring opening (Figure 5B). In acidic pH that stabilizes the lactone state of CPT the conjugation to ALOS4 did not accelerate the lactone ring opening (Figure 5A, 5C). Hence, CPT conjugation to ALOS4 through ring E increases CPT chemo-stability in physiological pH of 7.4.

### ALOS4-CPT conjugate induces DNA damage and cell death specifically in malignant melanoma cells

**Figure 6:**
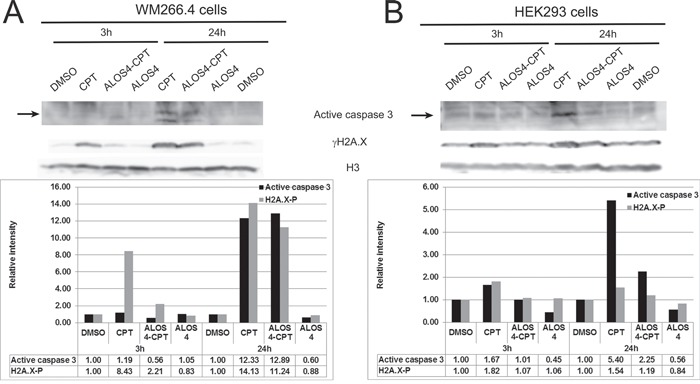
ALOS4-CPT conjugate specifically induces DSBs and caspase 3 activation in WM-266-4 cells Western blot analyses with antibodies recognizing γH2A.X, active caspase 3 and histone H3 of lysates from cells treated with the indicated drugs for 3 hours and 24 hours. In each lane the band intensities of γH2A.X and active caspase 3 were quantified with ImageJ and normalized to the relative amount of histone H3. The graphs show the relative band intensities. The sample of the 3 hours DMSO treated cells was set as 1. **A.** WM-266-4 human metastatic melanoma **B.** non-malignant HEK293 cells.

Topoisomerase I inhibition by CPT results in DNA double strand breaks (DSBs) due to collisions between replication forks and stalled Topoisomerase 1 [31] as well as in inhibition of replication due to prevention of DNA uncoiling [32]. It is thought that both mechanisms induce cell death [33, 34]. The successful internalization of ALOS4-CPT that is followed by nuclear accumulation of CPT (Figure 4) led to evaluate the toxicity of ALOS4-CPT. For that purpose we monitored the formation of DSBs by the levels of γH2A.X (a phosphorylated form of the histone isoform H2A.X) and the induction of cell death by the levels of active caspase 3. Addition of ALOS4-CPT to WM-266-4 human malignant melanoma cells for 24 hours resulted in ~11 and ~13-fold increases in the levels of γH2A.X and active caspase 3, respectively (Figure 6A). These changes were similar to the changes in the positive control treatment of CPT alone for 24 hours. At the shorter time point (3 hours) caspase 3 did not undergo activation in any treatment while an increase in γH2A.X was monitored in CPT-treated cells and to less extent in ALO4-CPT-treated cells (Figure 6A). To monitor the specificity of this treatment we repeated the experiment using the non-malignant HEK293 cells (Figure 6B). Interestingly, in HEK293 cells treatment with ALOS4-CPT did not lead to an increase in the levels of γH2A.X at both time points, while active caspase 3 levels were increased moderately only at the 24 hours' time point. On the other hand CPT treatment led to almost 2 fold increase in the levels of both markers at the 3 hours' time point and more than 5-fold increase in the levels of active caspase 3 at the 24 hours' time point (Figure 6B). Thus, fusion of CPT to ALO4 gives specificity to the CPT-induced intracellular damage.

**Figure 7:**
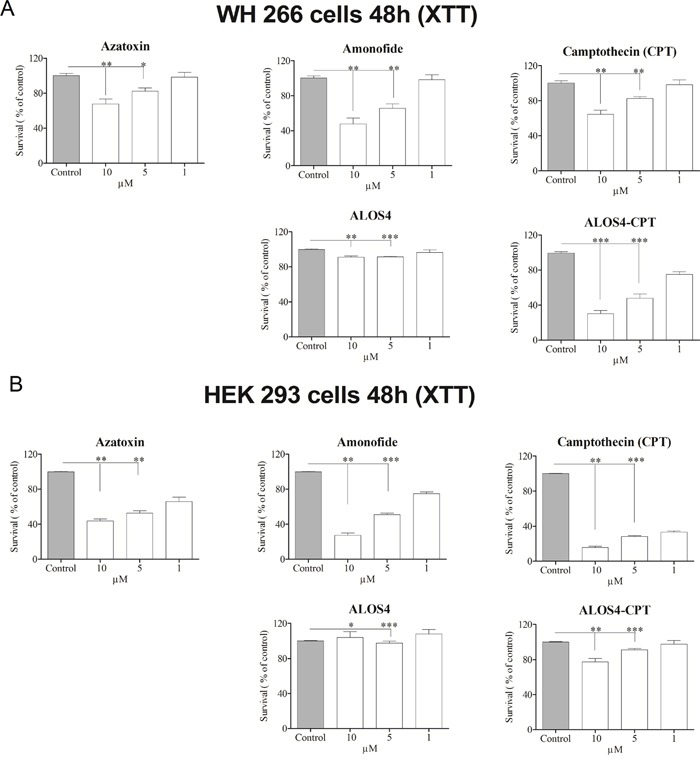
The effect of ALOS4-CPT conjugate on WM-266-4 cells growth The effect on cell growth of ALOS4-CPT *vs* the free anti-cancer drugs AM, AZA and CPT was studied by the XTT assay on **A.** WM-266-4 human metastatic melanoma and **B.** non-malignant HEK293 cells. The average relative amount of cells in comparison to control cells ±SD is presented. The statistical significance between different treatments was assessed using one-way ANOVA with post-hoc Bonferroni test, indicated by (*) at *p*<0.05, (**) at *p*<0.01 and (***) at *p*<0.001.

To evaluate whether ALOS4-CPT intracellular damage results in cell death we monitored the amount of viable cells following 48 hours treatment of ALOS-CPT as well as of CPT alone and two additional anti-cancer drugs: Azatoxin (AZA) and Amonafide (AM). Treatment of WM-266-4 cells with 10μM of ALOS4-CPT resulted in reduction of cell survival to 30% of the control cells, while treatments with a similar concentration of CPT, AZA and AM resulted in reductions in cell survival to 65%, 68% and 48% of the control cells, respectively. In agreement with previous results from mouse melanoma cells [25] ALOS4 by itself reduced the survival rate of WM-266-4 very moderately to 91% of the control cells (Figure 7A). To monitor the specificity of ALOS4-CPT treatment, we repeated the experiment using the non-malignant HEK293 cells. As expected, CPT, Aza and AM reduced significantly the survival rate of the cells to 16%, 44% and 27% of the control cells, respectively. However, fusion of CPT to ALOS4 resulted in a relatively moderate reduction in the survival rate of the cells to 77% of the control cells (Figure 7B).

### Modeling of ALOS4 binding to integrin α_v_β_3_

**Figure 8:**
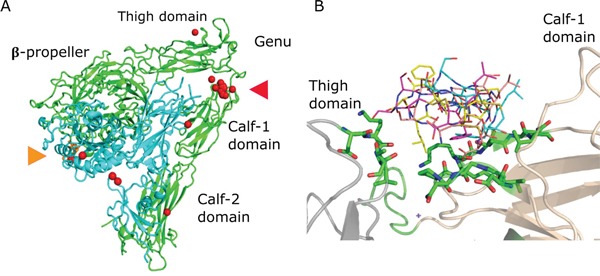
ALOS4 / integrin αvβ3 best twenty docking results **A.** The α_v_β_3_ extracellular segment (PDB code 1L5G) is shown using cartoon representation, the α_v_ and β_3_ chains are colored green and cyan, respectively. The RGD ligand is colored orange and shown using stick representation (marked with orange arrowhead). The best twenty ALOS4 docked position are shown as red spheres each sphere represent the center of the cyclic peptide. Twelve of twenty best results clustered at the interface between the Thigh and Calf-1 domains (marked with red arrowhead). **B.** Four representative ALOS4 docked peptides at the Thigh (gray) - Calf-1 (wheat) interface are showed using line representation. Two stretches of amino acids form the binding site, D550 – T553 (Thigh domain) and D682 – L693 (Calf-1 domain) are shown as sticks.

To gain insight regarding the possible mechanism of binding of ALOS4 to integrin α_v_β_3_ we used protein-peptide docking simulations using the Rosetta software [35]. For the simulations the receptor structure of the α_v_β_3_ that has been determined experimentally by Xiong *et al.* [36] was used and the 3D structure of the ALOS4 peptide was predicted computationally using the PEP-FOLD 1.5 server [37]. Overall, 10000 global docking trials were calculated between ALOS4 (ligand) and α_v_β_3_ integrin (receptor) in order to exhaustively explore all possible binding possibilities. The simulation results were ranked according to the binding energy and the twenty lowest energy ones (best binding modes) were further analyzed. Interestingly, twelve out of the best twenty docked positions cluster at the interface between the Thigh domain and the Calf-1 domain of α_v_ below the Genu (Figure 8A, red arrowhead). At this region the α and β integrin tails fold back at a ~135° angle, forming a V-shaped structure [36]. Whereas, only two of the best twenty docked positions localize to the RGD binding site (Figure 8A, orange arrowhead). Two stretches of amino acids form the binding site, D550 – T553 (Thigh domain) and D682 – L693 (Calf-1 domain) are shown as sticks (Figure 8B).

## DISCUSSION

Integrin α_v_β_3_ is considered a possible target for cancer therapy since it is over-expressed in several types of cancer such as melanoma, prostate cancer and breast cancer. In the past years vast efforts were made to develop specific and safe clinical tools to target integrin α_v_β_3_ for cancer therapy [23]. However, to date no such drug has reached the market. Here we tried to target integrin α_v_β_3_ by ALOS4, a newly discovered short and cyclic peptide [25]. Unlike the various integrin α_v_β_3_ binding peptides that have been developed so far, ALOS4 does not contain an RGD motif. Indeed our simulation predicts that ALOS4 docking site in integrin α_v_β_3_ is not the RGD binding site, but the twisted region of the integrin, which is mainly exposed while the integrin is in its bent conformation (Figure 8). According to most current models of integrin function, when inactive, the integrin headpiece is in a bent conformation pointing towards the cell membrane. In this conformation the RGD binding domain has low affinity for ligands. Upon activation by both intracellular proteins and extracellular ligands, a conformational change is induced that shifts the integrin to an extended conformation with an increased binding affinity to ligands [38]. We hypothesize that the unique binding site of ALOS4 may prevent integrin activation upon the peptide binding.

Apparently, ALOS4 binding to integrin α_v_β_3_ is strong enough to enable its accumulation on α_v_β_3_ highly expressing cells such as the human melanoma cell line WM-266-4 both *in vitro* and even *in vivo* in a xenograft model (Figure 1–2). Moreover, ALOS4 binding to integrin α_v_β_3_ enables its internalization along with CPT coupled to it that is followed by nuclear accumulation of CPT (Figure 4). CPT accumulation in the nucleus results in DNA damage and induction of cell death (Figure 6–7). Hence, coupling of CPT to ALOS4 does not interfere with CPT cytotoxic effects, while contributing a specific targeting tool to integrin α_v_β_3_ highly expressing cells, namely cancer cells.

Still, the anti-cancer cytotoxic effects of ALOS4-CPT are dependent on the stability of the conjugate in hemolytic and proteolytic environments. In this respect, the chemical instability of CPT due to the lactone ring opening that leads to its deactivation is a major obstacle [34]. Several publications reported on various methods for enhancement of lactone stability of CPT, all based on slight structural modifications like in the E-ring of Homocamptothecin [39] and linkage of CPT to glucuronide [40]. Here we conjugated ALOS4 to CPT through ring E in CPT, a linkage that slows down the lactone ring opening in basic physiological pH (7.4) (Figure 5). Thus, ALOS4-CPT is more stable than free CPT potentiating its utilization in TDD. Overall, our data suggest that ALOS4 can become a targeted carrier of cytotoxic drugs for treatment of human melanoma.

## MATERIALS AND METHODS

### Cell lines

WM-266-4 human metastatic melanoma cell line and HEK 293 cell line were cultured in RPMI medium (R8758, Sigma, Rehovot, Israel) supplemented with 2 mM glutamine (G7513, Sigma, Rehovt, Israel), 10% fetal bovine serum (F7524, Sigma, Rehovot, Israel) and penicillin and streptomycin (100 IU/ml each) (P4333, Sigma, Rehovot, Israel).

### Solid phase synthesis of ALOS4 conjugates

#### General information

CPT, Fluorescein isothiocyanate, all protected amino acids, resin and coupling reagents were purchased from Tzamal D-Chem Laboratories Ltd, Petah-Tikva, Israel. Azatoxin, [41, 42] and Amonafide [30] were synthesized according to literature procedures. All the solvents were purchased from Bio-Lab Ltd. Jerusalem, Israel or Gas Technologies Ltd. Kefar Saba, Israel. All other chemicals were purchased from Holland Moran or Sigma-Aldrich.

The synthesis of the cyclic peptides was done by a previously described procedure [43–48]. Briefly, 2-chlorotrityl chloride resin (1.12 mmol/gr) was placed in a reactor and suspended in DCM under nitrogen atmosphere. Then a mixture of Fmoc-Cys(Acm)-OH (2 eq.) and DIPA (8 eq.) in DCM was added. The resin loading reaction was allowed to proceed for 4-5 hr and then the resin was capped by an addition of a few drops of methanol. The Fmoc protecting group was removed with 20% piperidine/DMF (3 × 7 min) and then a linear SPPS was applied using standard Fmoc procedures introducing the AA in the following order: Fmoc-Phe-OH, Fmoc-Leu-OH, Fmoc-Ser(*t*-Bu)-OH, Fmoc-Gly-OH, Fmoc-Ala-OH, Fmoc-Ser(*t*-Bu)-OH, Fmoc-Cys(Acm)-OH and Fmoc-GABA-OH (MS Spectra is shown in Supplementary Figure S2). All the couplings were performed in DMF with 3-fold excess of AA and 6 eq. of DIPA, using PyBop for activation. Each coupling cycle was conducted for 2-3 hr. The completion of each coupling reaction and Fmoc removal were monitored by the ninhydrin test. After the coupling of the last AA, the sequence was cyclized by I_2_, washed and N-terminus Fmoc was deprotected yielding the cyclic peptidyl residue ready for drug conjugation. The scrambled peptide was synthesized using the same method as described previously, while randomly replacing the first 4 amino acids at the C terminus.

#### Synthesis of ALOS4 conjugates to FITC and CPT

The peptidyl resin was washed and a DCM solution of FITC and DIEA (FITC (2 eq.), DIEA (8 eq)) or a DMF/DCM (1:1) solution of premade 4-nitrophenylcarbonate derivative of CPT (anticancer agent (1.2 eq.), 4-nitrophenyl chloroformate (1.2 eq.), DMAP (1.2 eq.), DIPA (3 eq.) in DCM, 3h, rt) [49] were added correspondingly to the exposed primary amine for overnight at rt. The resin was thoroughly washed and the peptide conjugates were cleaved from the polymeric support with the cold TFA cocktail (95% TFA, 2.5% TIS, 2,5% H_2_O). The solvents were removed under a gentle flow of N_2_ and then the crude was precipitated from Et_2_O. Purification on semi-preparative HPLC by the method mentioned above provided the final conjugates to FITC and CPT, respectively. For the FITC conjugate: (42% yield, purity 95%)LC-MS:RT = 8.66 min;MS: ESI-MS m/z calcd: 1345.41 found: 673.8 (M2H^++^/2), 684.7 (MHNa^++^/2), 692.8 (MHK^++^/2) (Supplementary Figure S3). For the CPT conjugate: (34% yield, purity 93%) LC-MS: RT = 9.99 min; MS: ESI-MS m/z calcd: 1330.46 found: 666.3 (M2H^++^/2), 677.3 (MHNa^++^/2), 685.1 (MHK^++^/2) (Supplementary Figure S4).

### High performance liquid chromatography (HPLC)

All HPLC purifications were done via reverse phase on ECOM semi-preparative system with dual UV detection at 254 and 214 nm. Phenomenex Gemini® 10 μm C18 110 Å, LC 250 × 21.2 mm prep column was utilized. The column was kept at room temperature. The eluent solvents were 0.1% TFA in H_2_O (A) and 0.1% TFA in ACN (B). A typical elution was a gradient of 100% A to 50% B over 45 min at a flow rate of 25 mL/min. Analytical RP-HPLC was performed on an UltiMate 3000 system (Dionex) using a Vydac C18 column (250 × 4.6 mm) with 5 μm silica (300 Å pore size). Linear gradient elution (0 min 0% B; 5 min 0% B; 50 min 90% B) with eluent A (0.1% TFA in water) and eluent B (0.1% TFA in acetonitrile: H_2_O (80:20, v/v)) was used at a flow rate of 1 mL/min.

### Liquid chromatography - mass spectrometry (LCMS)

Electron spray mass spectra (ESI-MS) were obtained using an Autoflex III smart-beam (MALDI, Bruker), Q-TOF micro (Waters) or an LCQ Fleet™ ion trap mass spectrometer (Finnigan/Thermo). HPLC/LC-MS analyses were made using Agilent infinity 1260 connected to Agilent quadruple LC-MS 6120 series equipped with ZORBAX SB-C18, 50 × 2.1 mm, 1.8 μm column. In all cases the eluent solvents were A (0.1% TFA in H_2_O) and B (0.1% TFA in ACN) and the elution gradient profile was: 100% A for first 4 min, 8 min (from min 4 to min 12) during which it reached 100% B, 4 min (from min 12 to min 16) of 100% B, 2 min (from min 16 to min 18) during which it returned back to A, and 2 min (from min 18 to min 20) of 100% A. The UV detection was at 254 nm. Column temperature was kept at 50°C. The flow rate was of 0.4 ml/min. The MS fragmentor was tuned on 100V on positive or negative mode.

### Chemo-stability

0.1 mM CPT and ALOS4-CPT were incubated in acetate buffer at pH 4.5 and in PBS at pH 7.4 at 37°C. During the incubation period (24h - 144h), aliquots were removed at different time intervals, filtered and analyzed by LC-MS.

### FACS analysis

For evaluating integrin α_v_β_3_ expression and ALOS4-FITC binding the cells were washed with PBS and scrapped from the culture flask. 10^6^ of cells were incubated with 20 μL of ready for use FITC conjugated mouse anti-human CD51/CD61 antibody that specifically binds α_v_β_3_ integrin (#555505, BD Bioscience, San Jose, CA, USA) or with ALOS4-FITC at the indicated concentrations with or without a competitor scrambled peptide at 4°C for 45 min for the antibody or 2 hours for the peptides. Following two washings with PBS the cells were re-suspended in 400 μL of PBS and analyzed with Becton Dickson FACSCalibur cell analyzer equipped with an argon-ion laser (15W) at 488 nm with a 530/30 DF filter. For each sample ~ 10^4^ cells were analyzed. FlowJo software was used to analyze the collected data. For background measurement the cells were treated at the same conditions but without adding the antibody or the FITC-labeled peptide.

### Fluorescent and confocal microscopy

Fluorescent images of ALOS4-FITC were acquired by Photometrics CoolSNAP HQ2 camera mounted on an Olympus iX81 fluorescent microscope. For confocal microscopy analysis the cells were transfected with a plasmid expressing GFP-fused histone H1E (pH1E-GFP) [50] using Xfect polymer (ST0152, Clontech Laboratories, CA, USA) and were stained with CytoPainter Lysosomal Staining Kit - Red Fluorescence (ab112137, Abcam, Cambridge, MA, USA). Images were collected with a Zeiss LSM700 confocal microscope.

### *Ex vivo* fluorescent imaging

Five-week-old athymic nude mice (Harlan Labs, Nes Ziona, Israel) were subcutaneously inoculated in the dorsal left side with WM-266-4-cells, and tumors allowed to establish over time. When tumor volume reached 100 mm^3^, ALOS4-FITC (0.3 mg/kg) was intravenously administered. After 3 and 24 hours, the delivery efficiency of ALOS4-FITC was assessed using a Maestro™ fluorescence imaging system (n=5 in each group).

### Western blot analysis

The cells were scraped and washed with PBS buffer. Cell lysates were prepared by sonication in 1 X SDS sample buffer supplemented with protease inhibitor cocktail (MBS539134, Millipore) and boiling for 10 min. The proteins were separated by SDS-PAGE and transferred to nitrocellulose membranes. The following antibodies were used for western blotting: active caspase 3 (C8487, Sigma), γH2AX (05-636, Millipore) and histone H3 (05-928, Millipore). The band intensities in a representative experiment were quantified by the ImageJ program.

### Cell survival test

The cytotoxicity of the compounds was determined by measuring the mitochondrial enzyme activity, using a commercial XTT assay kit (#20-30-100, Biological Industries, Beit-Haemek, Israel) according to the manufacturer protocol using 2×10^3^ cells/well of a 96–well plate. The cells were cultured for 24 hours in growth medium followed by additional 48 hours with growth medium supplemented with different concentrations of the tested substances. The absorbance of the XTT reagent was measured with a TECAN Infinite M200 ELISA reader. The absorbencies were normalized to the absorbance of cells grown in medium and solvent. All the tests were repeated three times in quadruplicates.

### ALOS4 docking simulations to integrin α_v_β_3_

The ALSO4 cyclic peptide (ligand) was docked to integrin α_v_β_3_ (receptor). During each docking simulation the receptor remains stationary, while the ligand explores all six degrees of freedom (three translations and three rotations). The 3-dimentional structure of ALOS4 was predicted computationally using the PEP-FOLD 1.5 server [37]. The crystal structure of the extracellular segment of integrin α_v_β_3_ (Protein Data Bank entry 1JV2 [36]) was used as the receptor. Simulations of unbiased (global) docking between a peptide and the α_v_β_3_ receptor to explore all possible binding sites were performed using the Rosetta software [35] while allowing side chains flexibility.

## SUPPLEMENTARY FIGURES



## Abbreviations

ACN: acetonitrille
CPT: Camptothecin
CTLA-4: Cytotoxic T-Lymphocyte Antigen-4
DCM: dichloromethane
DIPA: diisopropylethylamine
DMAP: 4-*N,N*-(dimethylamino)pyridine
DMBA: dimethyl barbituric acid
DMF: *N,N*-dimethylformamide
DMSO: dimethyl sulfoxide
Fmoc: 9-fluorenylmethoxycarbonyl
HPLC: high-pressure liquid chromatography
LC-MS: liquid chromatography-mass spectroscopy
MALDI: matrix-assisted laser desorption/ionization
MAPK: mitogen-activated protein kinase
NCCN: National Comprehensive Cancer Network
PD-1: Programmed cell death protein 1
PD-L1: Programmed cell death 1 ligand 1
PyBOP: benzotriazol-1-yl-oxytripyrrolidino-phosphonium hexafluorophosphate
rpm: rounds per minute
SPOS: solid phase organic synthesis
SPPS: solid phase peptide synthesis
TFA: trifluoroacetic acid
TIPS: triisopropylsilane
XTT: 2,3-bis(2-methoxy-4-nitro-5-sulfophenyl)-5-[(phenylamino) carbonyl]-2H-tetrazolium hydroxides
