# Supplementary material for: Toward the development of a novel non-RGD cyclic peptide drug conjugate for treatment of human metastatic melanoma
Source: Oncotarget. 2016 Oct 19;8(1):757–68. doi: 10.18632/oncotarget.12748 (PMC5352194; doi:10.18632/oncotarget.12748)
Supplement: Supplementary file 1 [file oncotarget-08-757-s001.pdf]

Toward the development of a novel non-RGD cyclic peptide drug conjugate for treatment of human metastatic melanoma

SUPPLEMENTARY FIGURES

FACS ANALYSIS:

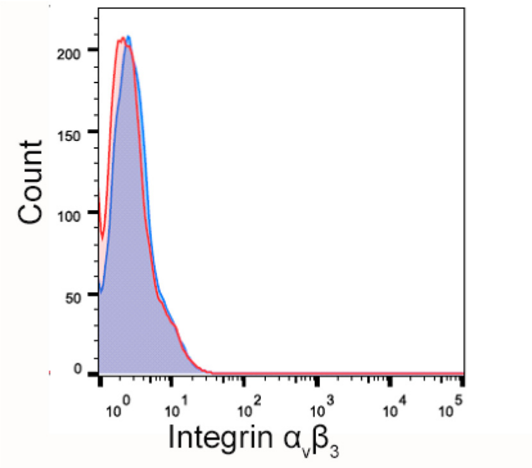

Supplementary Figure S1: Expression of integrin αvβ3 in HEK293 cells was evaluated by FACS analysis using FITC-conjugated integrin αvβ3 antibody (red- control cells w/o an antibody, blue- cells incubated with FITC-conjugated integrin αvβ3 antibody).

SPECTRA:

ALOS4
HRMS:

Qualitative Compound Report

Compound Table

Compound Label	RT	Mass	Abund	Formula	Tgt Mass	Diff (ppm)
Cpd 1: C35 H53 N9 O13 S2	0.133	871.3193	2411	C35 H53 N9 O13 S2	871.3204	-1.3

Compound Label	m/z	RT	Algorithm	Mass
Cpd 1: C35 H53 N9 O13 S2	894.3097	0.133	Find By Formula	871.3193

MS Spectrum

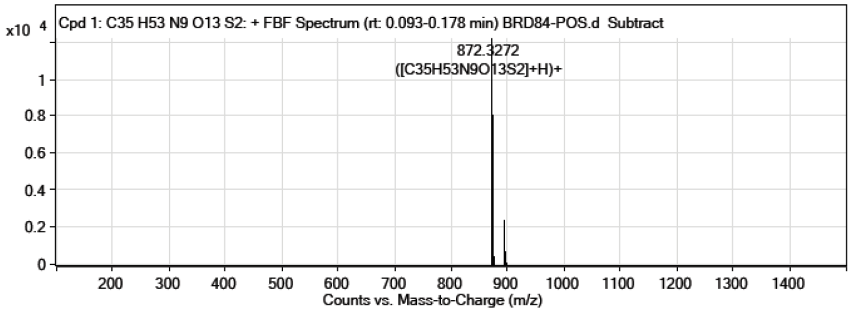

MS Zoomed Spectrum

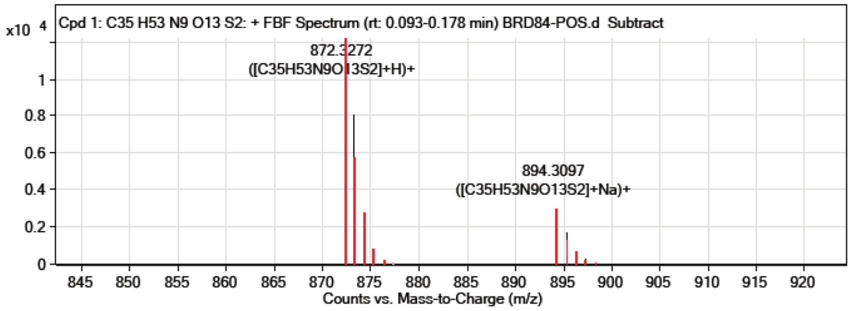

MS Spectrum Peak List

m/z	z	Abund	Formula	Ion
872.3272	1	12169.84	C35H53N9O13S2	(M+H)+
873.3288	1	8004.44	C35H53N9O13S2	(M+H)+
874.3279	1	1694.47	C35H53N9O13S2	(M+H)+
875.3329	1	408.91	C35H53N9O13S2	(M+H)+
876.3216	1	194.63	C35H53N9O13S2	(M+H)+
894.3097	1	2410.55	C35H53N9O13S2	(M+Na)+
895.3094	1	1701.5	C35H53N9O13S2	(M+Na)+
896.3069	1	601.07	C35H53N9O13S2	(M+Na)+
897.3059	1	240.32	C35H53N9O13S2	(M+Na)+
898.3025	1	94.4	C35H53N9O13S2	(M+Na)+

--- End Of Report ---

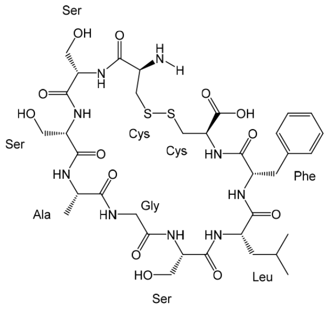

ALOS4
Chemical Formula: $C_{35}H_{53}N_9O_{13}S_2$
Exact Mass: 871.32
Molecular Weight: 871.98

Figure 2: ALOS4.

ALOS4-FITC
ES-MS:

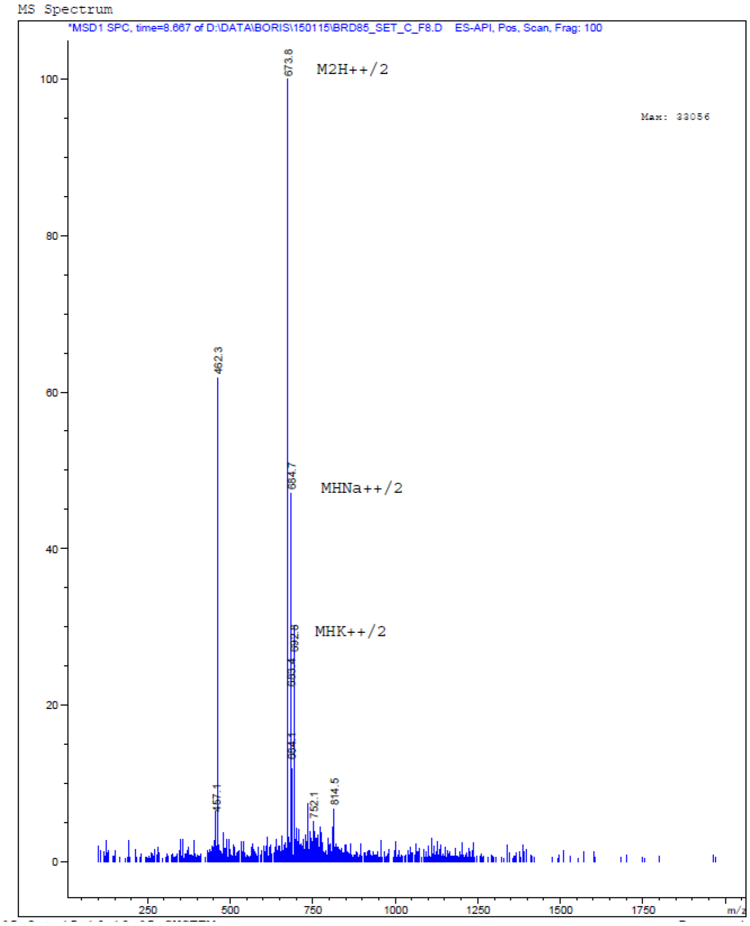

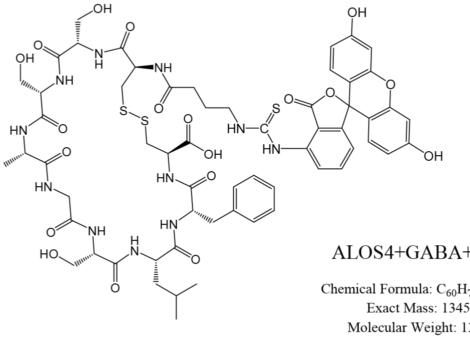

ALOS4+GABA+FITC

Chemical Formula: $C_{60}H_{71}N_{11}O_{19}S_3$
Exact Mass: 1345.41
Molecular Weight: 1346.47

Figure 3: ALOS4-FITC.

ALOS4-CPT
ES-MS:

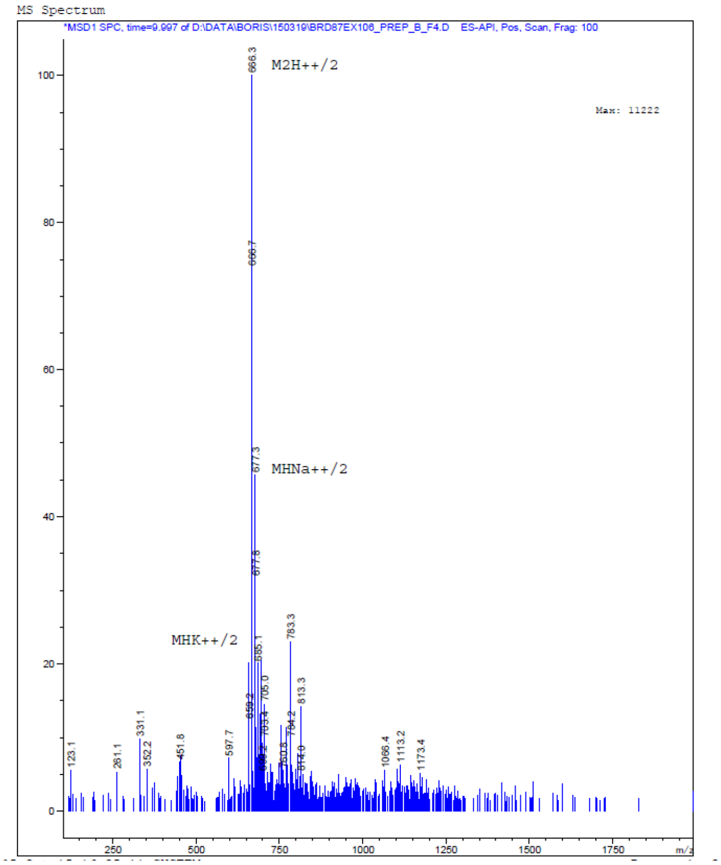

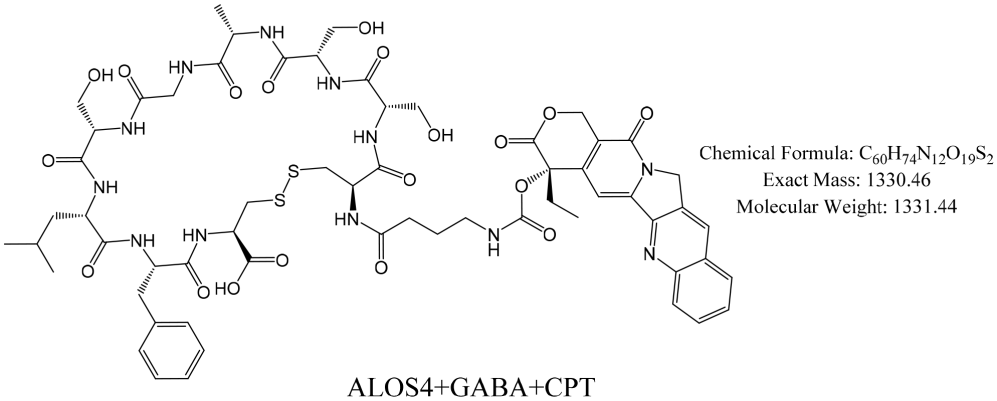

Chemical Formula: $C_{60}H_{74}N_{12}O_{19}S_2$
Exact Mass: 1330.46
Molecular Weight: 1331.44

ALOS4+GABA+CPT

Figure 4: ALOS4-CPT.
